# Combined low-carbohydrate diet and long-term exercise in hypoxia in type 2 diabetes: A randomized controlled trial protocol to assess glycemic control, cardiovascular risk factors and body composition

**DOI:** 10.1177/02601060231190663

**Published:** 2023-07-27

**Authors:** Raquel Kindlovits, Ana C Sousa, João L Viana, Jaime Milheiro, Franklim Marques, Vitor H Teixeira

**Affiliations:** 1 Faculty of Nutrition and Food Sciences, 26706 University of Porto, FCNAUP, Porto, Portugal; 2 Research Center in Sports Sciences, Health Sciences and Human Development, CIDESD, University of Maia, Maia, Portugal; 3 CMEP, Exercise Medical Center, Porto, Portugal; 4 Laboratory of Biochemistry, Department of Biological Sciences, Faculty of Pharmacy, 26706 University of Porto, FFUP, Porto, Portugal; 5 Research Center in Physical Activity, Health and Leisure, CIAFEL – Faculty of Sports, 26706 University of Porto, FADEUP, Portugal; 6 Laboratory for Integrative and Translational Research in Population Health, ITR, Porto, Portugal

**Keywords:** Diabetes, carbohydrates, training, hypoxia, insulin, body composition

## Abstract

**Background:** Cardiovascular disease is the leading cause of mortality associated with diabetes, which is characterized by chronic hyperglycemia. Low-carbohydrate diet has gained popularity as an intervention in patients with type 2 diabetes mellitus, acting to improve glycemic profile and serum lipids. In its turn, exercise in hypoxia induces specific adaptations, mostly modulated via hypoxia-induced transcription factor signaling cascade, which increases with exposure to altitude, and promotes angiogenesis, glycogen supply, glucose tolerance, and raises GLUT-4 expression. **Aim:** Given that hyperglycemia decreases HIF-1α and it is better controlled when following a low-carbohydrate diet, this study aims to examine the hypothesis that a combination of both low-carbohydrate diet and chronic exercise in hypoxia in type 2 diabetes mellitus is associated with improved glycemic control and cardiovascular parameters, whose protocol is described. **Methods:** Patients with type 2 diabetes mellitus (*n* = 48) will be recruited and randomized into one of the three groups: (a) Control group: Control diet (low-fat and moderate-carbohydrate diet) + exercise in normoxia; (2) exercise in hypoxia group: Control diet + exercise in hypoxia; (3) intervention group: Low-carbohydrate diet (low-carbohydrate and high-fat diet) + exercise in hypoxia. Before and after 8 weeks of interventions, cardiopulmonary tests (Bruce protocol), body composition and blood pressure will be evaluated. Blood samples will be collected to measure hypoxia-induced transcription factor, C-reactive protein, glycemic and lipid profiles. **Summary:** This will be the first trial to examine the isolated and combined effect of chronic exercise in hypoxia and low-carbohydrate diet in type 2 diabetes mellitus. This trial will help to fill a significant research gap, guide future research and contribute to the combined nutrition and exercise approach to type 2 diabetes mellitus.

## Introduction

The worldwide prevalence of type 2 diabetes mellitus (T2DM) in adults has increased from ∼150 million people in 2000 to >450 million in 2019, and is projected to rise further to ∼700 million by 2045 (Ogurtsova et al., 2022). Genetics and poor lifestyle behaviors, such as consumption of a high-sugar diet and a sedentary lifestyle, can have a predisposing influence on T2DM, which occurs at varying rates in people of different racial and/or ethnic backgrounds (Zhu et al., 2019). By definition, T2DM describes a group of metabolic disorders characterized by high blood glucose levels. People with T2DM are at an increased risk of developing a number of serious, life-threatening health problems, resulting in higher medical care costs, reduced quality of life, and increased mortality, mainly from cardiovascular diseases (Baena-Diez et al., 2016; Kelsey et al., 2022).

It is consensual that diet is the key strategy for preventing and managing T2DM. However, controversy still exists about the best dietary approach, namely the optimal macronutrient composition. A low-carbohydrate diet (LCD) has been proposed as a nutritional strategy with advantages in controlling glucose homeostasis and improving cardiovascular disease risk factors (Evert et al., 2019). Several studies have demonstrated improvements in markers of glycemic control and insulin sensitivity (Elhayany et al., 2010; Jenkins et al., 2018; Miyashita et al., 2004), and in serum lipids (Davis et al., 2009; Elhayany et al., 2010; Kirk et al., 2008) with broad-spectrum carbohydrates LCD (from 21 g daily up to 45% of daily energy intake). In addition to a healthy diet, exercise has been considered a cornerstone of T2DM therapy for many years (Colberg et al., 2016; Joslin, 1916). It is well established that regular physical activity is of paramount importance for the management of T2DM, as it has beneficial effects on the cardiometabolic risk factors involved in the development of diabetes complications (American Diabetes, 2000).

Exercise in hypoxia (EH) has recently gained attention from the scientific community as a valuable therapeutic strategy (Millet et al., 2016). Actually, EH has been associated with health benefits in chronic obstructive pulmonary disease (Haider et al., 2009), coronary artery disease (Burtscher et al., 2004) and obesity (Urdampilleta et al., 2012). Chronic EH has been shown to influence some important mechanisms related to glycemic control, such as increasing hypoxia-induced transcription factor (HIF-1α), the activity of oxidative enzyme, glycogen supply, glucose tolerance and GLUT-4 expression (Faramoushi et al., 2016). These specific adaptations induced by hypoxia raise the question about the usefulness of EH as a therapeutic intervention in T2DM, but the evidence is still scarce.

HIF-1α appears to be a key element in the EH-induced adaptations, regulating the expression of genes associated with angiogenesis, such as vascular endothelial growth factor, vascular endothelial growth factor (VEGF) (Thangarajah et al., 2010). The low oxygen availability in EH promote the production of HIF-1α to a greater extent than when training under normoxia (sea-level) conditions (Desplanches et al., 1993). On the other hand, a high glucose environment impairs the activity of the HIF-1α. Thus, it is theoretically possible that a combined effect of LCD and EH works synergistically to improve T2DM, compared to the single effect of EH intervention, but the effectiveness of this hypothesis is currently unknown.

This article describes the protocol of a randomized, single-blind and parallel-controlled clinical trial that will evaluate the effects of chronic (8 weeks) EH, alone or combined with an LCD, on glycemic control and cardiovascular risk factors in patients with T2DM. The primary outcome of this randomized clinical trial (RCT) is to determine the effect of these interventions on hemoglobin A1c, while the secondary aims are to assess the impact on fasting blood glucose, plasma insulin and Homeostatic Model Assessment for Insulin Resistance (HOMA-IR), HIF1-α, cardiovascular risk factors and body composition. Due to a lack of well-conducted clinical trials, this study will provide original insights and important practical outcomes about EH and LCD interventions as non-pharmacological treatment modality in T2DM.

This study aims to examine the hypothesis that a combination of both LCD and chronic EH in patients with T2DM is associated with improved glycemic control and cardiovascular risk factors, whose protocol is described.

## Material and methods

This article has been written in accordance with the SPIRIT (Standard Protocol Items: Recommendations for Interventional Trials) and CONSORT (Consolidated Standards of Reporting Trials) guidelines (Chan et al., 2013).

### Study design and setting

This is an 8-week, controlled, single-blind, three-arm, parallel, RCT, which will take place at the Faculty of Nutrition and Food Sciences and the Faculty of Sports of the University of Porto, at the Exercise Medical Center (CMEP) in Porto and at the Research Center in Sports Sciences, Health Sciences and Human Development (CIDESD) of the University of Maia. Participants will be randomly assigned into three independent groups (*n* = 16, in each) and the experimental design will consist of (a) pre-intervention tests, (b) familiarization period, (c) experimental intervention and (d) post-intervention tests.

### Eligibility criteria

To be included, participants must meet the following criteria: (a) Individuals of either sex diagnosed with type 2 diabetes by their medical doctor for at least one year; (b) hemoglobin A1c between 6.5% and 10%; (c) pharmacological regimen stabilized for at least three months; (d) previous participation in supervised exercise programs in the last 6 months, and (e) absence of smoking in the last 6 months. Participants will be excluded if: (a) They are insulin-dependent; (b) they have uncontrolled microvascular or macrovascular complications related to diabetes, such as retinopathy, nephropathy, diabetic foot and atherosclerosis, diabetic cardiomyopathy or acute myocardial infarction; (c) present other uncontrolled metabolic and vascular comorbidities; (d) are sedentary or (e) have physical limitation that prevent them from exercising. It is worth mentioning that medications, including doses and schedules, at baseline and changes throughout the study will be evaluated and documented.

### Recruitment

**Figure 1.**
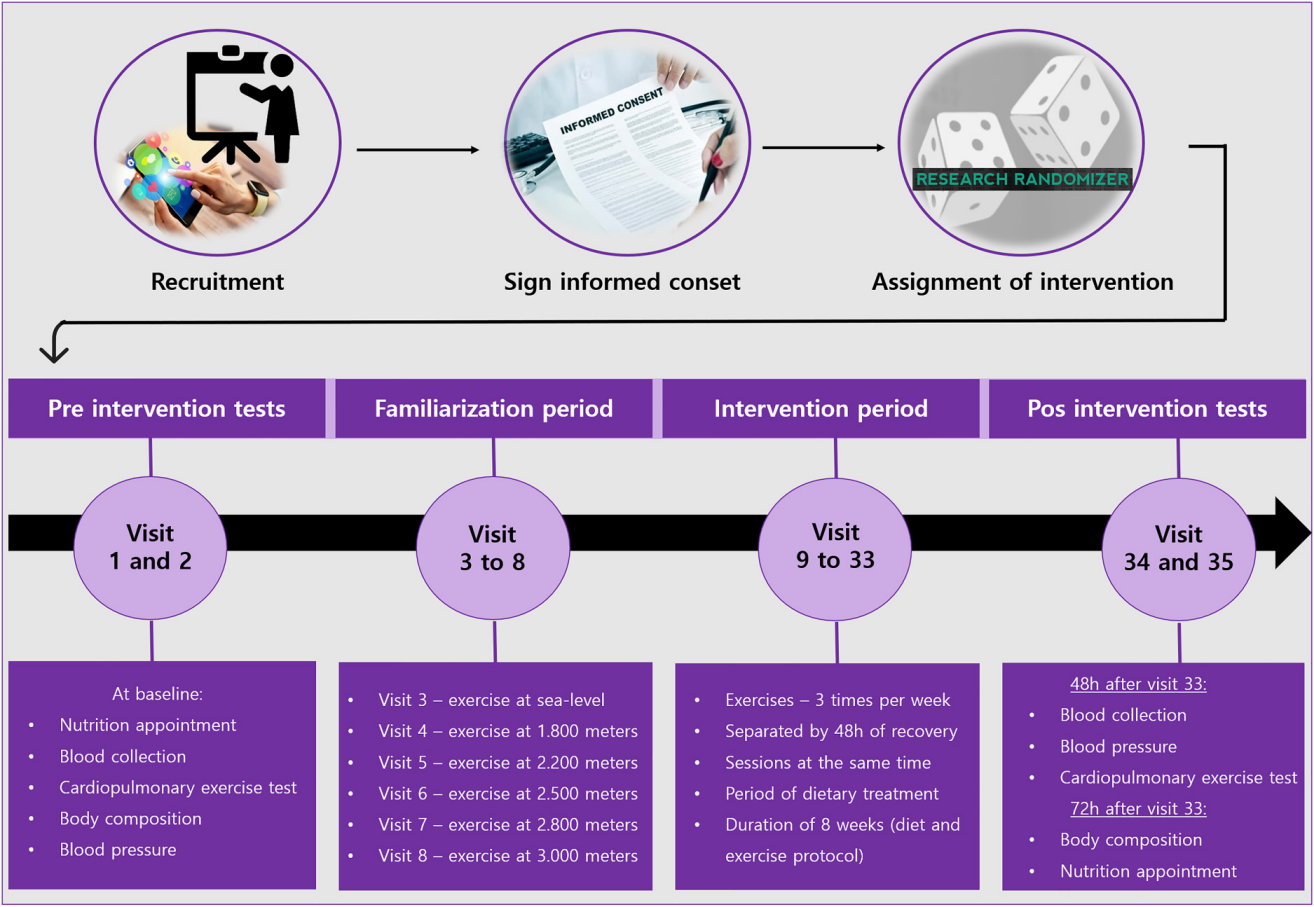
Schematic diagram of participant visits during the study.

The recruitment period for this study will end in September 2023. After sample size calculation and clinical trial registration, participants will be invited to participate in the study through meetings, electronic and social media. The participant's timeline is shown in Figure 1.

### Assignment of intervention

**Figure 2.**
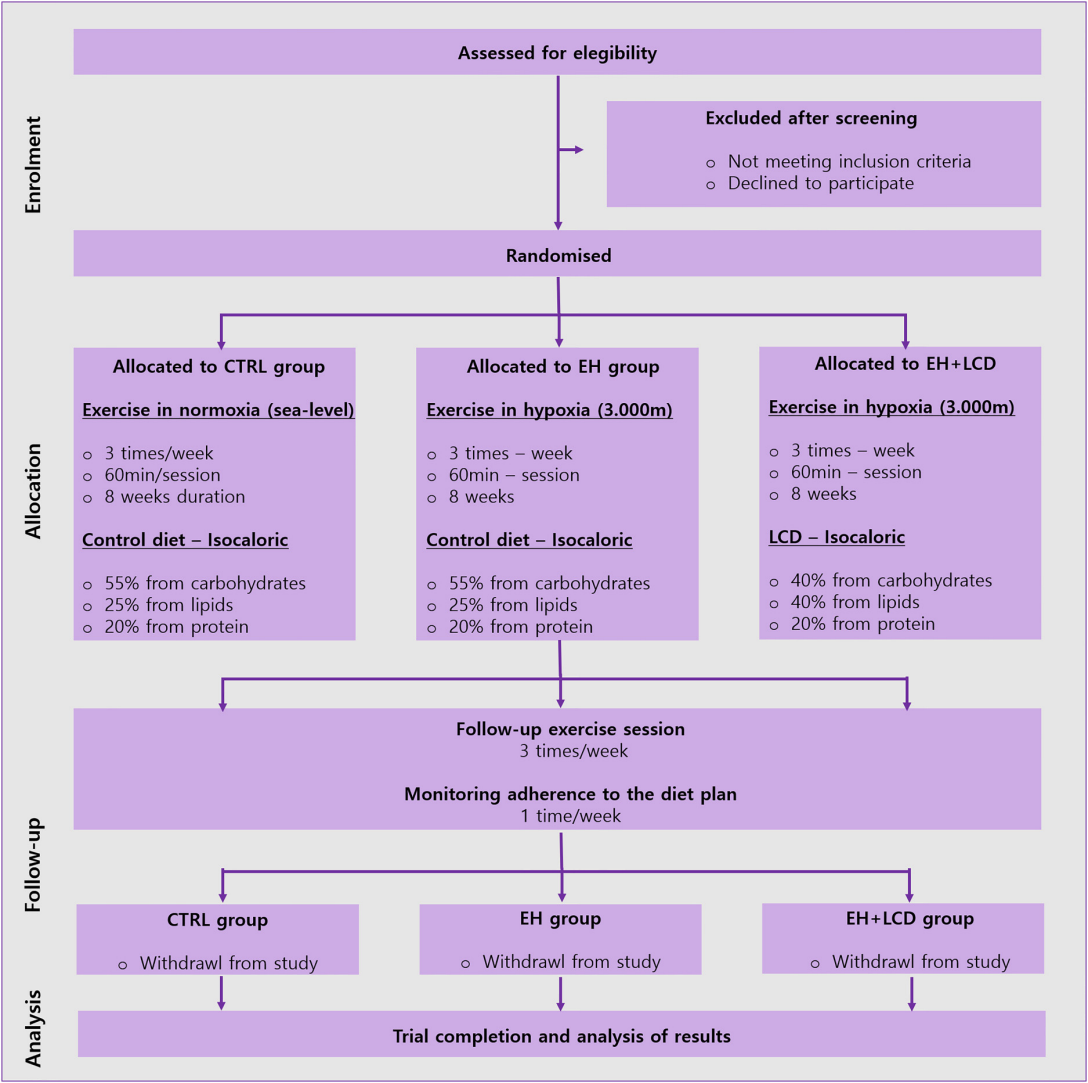
CONSORT flow diagram of the progress through the phases of the parallel randomized trial of three groups. CTRL: control group: EH: exercise in hypoxia; LCD: low carbohydrate diet.

Participants confirmed as eligible will be randomized at a ratio of 1:1:1 to each group in blocks of four participants until complete the total number calculated (*n* = 48). A computer-generated random numerical sequence will be used for randomizing participants (Research Randomizer). The principal researcher will generate the sequence and conceal it in sealed, numbered opaque envelopes. Participants will be allocated a sequential number upon screening. The sealed envelope corresponding to the allocated participant number will be opened on the first visit by the principal researcher, and the participants will be allocated, without knowing which group they will belong to. As this is a single-blind study, blinding is not required for principal researcher, only to participants. A Consolidated Standards of Reporting Trials (CONSORT) flow chart outlining the study phases is displayed in Figure 2 (Moher et al., 2001).

### Interventions

Volunteers who meet the inclusion criteria will be asked to sign a written informed consent before enrolment in the trial, with the principal researcher. Participants will be randomized into control (CTRL), EH or interventions (EH + LCD) groups during 8 weeks. The CTRL group will perform an exercise protocol in normoxia with a control diet, and the other groups will perform the same exercise protocol in hypoxia (simulated altitude – nitrogen dilution method), following a control diet (EH) or an LCD (intervention group). Thus, we will compare whether EH group is more efficient than exercise in normoxia (CTRL group) and, if by including a LCD (EH + LCD group) the effects of EH group would be potentiated. We emphasize that during all test, physical exercise professionals, nutritionist, and doctors or nurses will be available.

### Dietary plan

An individualized dietary plan will be prescribed for each participant, using the software Dietbox®, version 7.0. The energy content of the dietary plan will meet 100% of the energy requirement for each participant, which will be estimated by multiplying the resting metabolic rate obtained by the Harris–Benedict equation, the most accurate at an individual level for older adults (Cioffi et al., 2021), and by a physical activity level, assessed using the International Physical Activity Questionnaire (IPAQ short form, last 7 days, elderly, self-administered format). Participants will be categorically rated into one of three levels of physical activity – low, moderate or high (Lee et al., 2011).

The energy distribution by macronutrients of the control diet of the CTRL and EH groups will be 60% from carbohydrates, 20% from protein and 20% from fat. The LCD will consist in 40% of energy from carbohydrates, 20% from protein, and 40% from fat. Both diets will be based emphasis on low-glycemic index foods and will limit saturated fat to 10% of energy, to reflect conventional dietary guidelines (Rodbard et al., 2007). We use the classification of diets as (a) very low-carbohydrate: less than 26% of the energy intake; (b) mild low-carbohydrate: 26%–45% of the energy intake; and (c) high carbohydrate ≥ 45% of the energy intake (Silverii et al., 2020). Thus, we will compare a mild LCD (EH + LCD group) to a high-carbohydrate diet (EH group). Compliance with the dietary plan will be assessed using weekly 24-h recalls. Participants will meet individually with a nutritionist twice within 8-week period to encourage adherence to the dietary plan.

### Exercise protocol

Both exercises in normoxia and in normobaric hypoxia at 3.000 m of simulated altitude, will take place 3 times per week, during period intervention of 8 weeks, in a hypoxic chamber at CMEP – Exercise Medical Center, Porto – Portugal. The chamber allows to control O_2_ (11%–20.97%), temperature (until 50 °C), humidity (until 80%) and altitude (until 8000 m). Altitude rating can be defined as (a) high altitude: 1.500–3.500 m; (b) very-high altitude: 3.500–5.500 m and (c) extreme altitude: Above 5.500 m, over sea level (Mathew and Sharma, 2022).

Exercise sessions will consist of 45 min of moderate aerobic exercise on a cycle ergometer and treadmill (Life Fitness, Illinois, USA) and 15 min of strength training, as recommended for this population (Bull et al., 2020). Moderate exercise intensity, which is considered an efficient strategy to improve cardiorespiratory and metabolic health in T2DM (Gildea et al., 2021), will be defined using 75% of the heart rate reserve, quantified in the pre-intervention cardiopulmonary exercise test (CPET). During each exercise session, heart rate and oxygen saturation will be constantly monitored using a finger pulse oximeter (Globus YM201, Milan, Italy). All exercise sessions will take place at the same time of day (± 2 h) and visits will be separated by at least 48 h of recovery.

### Strategies to improve adherence

Participants will have the opportunity to obtain chauffeured transport to each session and assessment locations at no cost. A weekly session will be dedicated to clarifying doubts about the diet plan with a nutritionist, and it will also be possible to contact the nutritionist by telephone, in order to facilitate access and adherence to the dietary plan. A social media group will be created to facilitate routine communication and remind participants of scheduled tasks. Throughout the study, the research team will assess participant's motivation and limitations. It is noteworthy that all participants are aware of the opportunity to attend the only clinic in Portugal that allows exercising within a hypoxic environment, with multidisciplinary supervision and free of charge.

### Study outcomes

The main outcome will be hemoglobin A1c levels after 8 weeks of interventions. A set of secondary clinical outcomes will include complete blood count with hemoglobin and reticulocytes, fasting glucose, fasting insulin, HOMA-IR, total-, LDL- and HDL-cholesterol levels, triglycerides, C-reactive protein, serum HIF1-α levels, systolic and diastolic blood pressure, body composition, cardiorespiratory and pulmonary gas-exchange variables. These outcomes will be evaluated at baseline and at the end of the study (eighth week), in all groups.

### Biochemical analyses

Blood samples will be collected after fasting for 12 h, no strenuous exercise in the last 24 h, and alcohol consumption in the last 72 h. Glycemic profile, namely hemoglobin A1c (%), fasting insulin (Uui/mL) and fasting glucose, and cardiovascular risk factors, namely total-, LDL- and HDL-cholesterol, triglycerides and C-reactive protein levels will be measured and expressed in mg/dL. Also, Serum HIF-1α levels (pg/mL) will be measured using the HIF1A Human ELISA Kit (EHIF1A Thermo Fisher Scientific, Milan, Italy) according the manufacturer's protocols.

### Blood pressure

Systolic, diastolic, and mean blood pressure will be measured using an automated sphygmomanometer (Dinamap Pro; Florida, United States), in the left arm, in a sitting position and after 10 min of rest, on the same day of blood sample collection. The measurements will be in triplicate and the average will be used and expressed in mmHg.

### Body composition

A stadiometer with 0.1 cm intervals will be used to measure height, and a digital scale accurate to 0.1 kg, and a maximum capacity of 150 kg, will be used to evaluate weight (Seca, Hamburg, Germany). Waist circumference (WC) will be measured with an inelastic tape placed at the midpoint between the anterior-superior iliac crest and the last rib, as hip circumference (HC) will be measured at the widest portion of the buttocks, with the tape parallel to the floor (Norton, 2018). The ratio between WC and HC will be obtained and expressed in cm. Body mass index will be calculated by the ratio between weight and squared height and expressed in kg/m^2 ^(Norton, 2018). Lean mass, fat mass, android and gynoid fat depots, and bone mineral density will be assessed by dual-energy X-ray absorptiometry (DEXA; GE® Lunar densitometer, DPX NTVR, with ENCORE 2011 software, version 13.60, GE Healthcare). Participants will be instructed not to perform other procedures with contrast or radiation the day before the measurement. The procedure will be performed after 12 h of fasting, in the supine position, with bare feet, light clothes and no metallic objects.

### Cardiopulmonary exercise test

All participants will complete a standard Bruce treadmill exercise test protocol (Myers et al., 2002) before and 48 h after the last exercise session, using an electrocardiogram and a face mask device to evaluate gas exchange (Cortex Metalyzer 3B®, Leipzig, Germany). The initial stage of the exercise involves walking on the treadmill at a speed of 2.7 km/h with a 10% gradient. After 3 min, treadmill speed and gradient are increased, with further increments every 3 min until the end of the test. Criteria for termination will be volitional fatigue or when 85% of age-predicted maximal heart rate ((220 – age)/0.85) is attained, or if any medical indications arise, or upon participant request. Exercise tolerance, cardiac and ventilatory responses will be obtained and a report with clinical conclusions will be made.

CPET uses non-invasive exercise stress testing to generate multiple variables that are then extrapolated from this data, including anaerobic threshold and VO_2_max, markers of aerobic capacity. Anaerobic threshold will be defined as the point at which minute ventilation increases disproportionately relative to VO_2_, a response that is generally seen at 60%–70% of VO_2_max (Albouaini et al., 2007). In our study, peak oxygen consumption (VO_2_peak), minute ventilation (VE), respiratory exchange ratio, and metabolic equivalent value will be measured. Capillary blood samples (25 µL) for blood lactate will be collected from the fingertip before and immediately at the end of the test (Lactate Pro, Arkay, Inc., Kyoto, Japan), such as heart rate, blood pressure and oxygen saturation. Borg rating of perceived exertion (6–20 points) will be recorded at the end of CPET.

### Statistical considerations

For sample and power calculations, this study is powered based on changes in hemoglobin A1c in RCTs included in the meta-analysis by Zuuren et al. (2018), which tested the effect of carbohydrate restriction up to 8 weeks. Considering a type I error of 5% and a power of 80% to detect differences at the dependent variable with a statistical significance when comparing 3 independent groups (CTRL versus EH and EH versus EH + LCD), and an effect size of 1.14, a sample size of 14 participants per group will be needed, totaling 42 individuals. With an assumption of a 20% dropout rate, we will recruit 48 participants. The sample size calculations were performed using the Statistical Power Analyses for Windows, (G*Power 3.1.9.4, Uni Kiel, Germany).

Statistical analysis will be performed using SPSS statistics software version 28.0, 2021 (IBM Company, Chicago, United States). All data will be reported as mean ± SD and statistical significance will be assumed at *p* ≤ 0.05. Pairwise comparisons will be analyzed using the Wilcoxon test. A two-way repeated measures ANOVA will be used to examine changes in cardiopulmonary parameters, body composition, glycemic profile, and cardiovascular risk factors over the chronic exercise period (0 vs. 8 weeks), and whether the magnitude of the chronic exercise-mediated adaptations differ between groups (EH vs. CTRL; LCD + EH vs. EH). When a significant main- or interaction-effect will be found, Bonferroni post hoc tests for multiple pairwise comparisons will be performed to identify differences between groups.

### Data collection and management

A questionnaire will be applied in the first nutrition appointment, at the beginning of the study, to obtain the eating habits, socio-demographic and clinical characteristics of participants, including age, sex, marital status, schooling level, diabetes duration, family history of diabetes and self-reported physical activity. Drugs for T2DM or comorbidities and nutritional supplements will be registered at baseline and at the end of the study.

Blood samples will be collected and centrifuged at 1000 g for 10 min to separate plasma or serum, which will be stored at −80 °C for further analysis of biochemical parameters.

During the intervention period, the team of researchers will be trained to apply the 24-h food recall, constantly checked the oxygen saturation and supervise the maintenance of the heart rate at the target intensity of 75% of the heart rate reserve. All researchers will be trained to circumvent any cases of hypoglycemia, hypotension or possible side effects of high-altitude exposure, such as headache or difficulty breathing. Also, it will be qualitatively documented and details of each case of withdrawal and, the primary hypothesis will be tested on a superiority framework.

All data will be collected and stored according to Good Clinical Practice Guidelines (Switula, 2000). Data monitoring and auditing of the trial will be conducted by the principal researcher and supervisory team: V.H.T., J.L.V and A.C.S. All data, such as consent forms, questionaries, dietary screening forms, IPAQ and clinical data in general, will be stored in a secure database password-protected document that will be accessed only by authorized members of the research team. Dietary plans will be stored securely in Dietbox® Software, and all information will be treated confidentially. Data will only be used for the purpose of this research project.

### Ethical considerations

The study was approved by the Ethics Committee of the Faculty of Nutrition and Food Sciences, University of Porto (Approval Number 45/2021/CEFCNAUP/2021 – Supplementary Material) in July 2021, and will be conducted in accordance to the declaration of Helsinki for studies in human (World Medical, 2013). It has been registered in the Clinical Trial Database (NCT05094505), and any important protocol modifications will be recorded on it. The study strictly follows the protocols regarding informed consent form, confidentiality and anonymity. An informed consent form with guidelines, objectives of the project, description of procedures, possible risks and benefits will be provided. Participants will also be informed that participation is voluntary, and they can withdraw from the study at any time, without prejudice. In addition, if requested, participants can access their individual data at the completion of the trial by contacting the lead researcher.

### Dissemination policy

The results from this study will be published in a peer-reviewed and open-access academic journal or in an open-access institutional repository. Participants in the trial will also be notified of the outcomes.

## Summary

This RCT aims to compare the effects of 8 weeks of EH (simulated high-altitude at 3.000 m), combined or not with an LCD (40% of energy from carbohydrates) on glycemic and lipid profiles, HIF1-α, C-reactive protein, blood pressure, body composition and cardiorespiratory parameters in patients with T2DM. The metabolic benefits of the 8-week duration of both interventions alone have been previously demonstrated in T2DM (Boule et al., 2001; Maiorana et al., 2002; van Zuuren et al., 2018)

These non-pharmacological treatments (LCD and EH) can improve T2DM control and prevent chronic complications due to uncontrolled glycemia and impaired vascularization (Ladage et al., 2012; van Zuuren et al., 2018). Several authors have shown beneficial effects on T2DM in people who following a LCD, mainly in the short-term intervention (Sainsbury et al., 2018; Snorgaard et al., 2017). Sainsbury et al. (Sainsbury et al., 2018) showed that restricting carbohydrates to less than 26% of total energy results in greater reductions in hemoglobin A1c at 3 and 6 months than later (12 and 24 months). This may be due to the challenge of maintaining carbohydrate restriction, which is even more evident in very low carbohydrates diets, such as the ketogenic diet (Goldenberg et al., 2021). To circumvent this and improve dietary adherence, the present study assumed a lower carbohydrate restriction, consisting of 40% of the energy intake, which also has demonstrated benefits in metabolic control in patients with T2DM during 8 weeks (van Zuuren et al., 2018).

Also, the currently available studies have some methodological flaws, such as non-randomized or uncontrolled clinical trials (Kindlovits et al., 2022) and insufficient supervision of the reasons for non-adherence to prescribed LCDs (McArdle et al., 2019). These limitations make it difficult to translate their results into clinical practice.

Few studies have analyzed the effects of EH on glycemic and lipid profiles, on cardiorespiratory physiology or on angiogenesis in T2DM (Kindlovits et al., 2022). Furthermore, none of them evaluated HIF1-α levels, which is frequently destabilized in patients with T2DM (Thangarajah et al., 2010). Experiments on cells bearing inactivating mutations in the HIF pathway have emphasized the importance of HIF1-α on the regulation of genes involved in angiogenesis (Iyer et al., 1998; Krishnamachary et al., 2003). Considering that high glucose levels, common in patients with T2DM, impairs the activity of HIF-1α (Thangarajah et al., 2010), EH could be particularly beneficial to improve angiogenesis in this population, because the low oxygen availability in EH further enhances the production of HIF-1α to a greater extent than when training under normoxia conditions (Lundby et al., 2009; Robach et al., 2007). This in turn regulates VEGF expression (Li et al., 2019), appearing to be a key element in the EH-induced adaptations.

This study presents some novelties that make it stronger, such as: (a) it is the first study that will evaluate the serum levels of HIF1-α in T2DM, before and after exercise in normoxia and in hypoxia, with or without a restricted carbohydrate intake; (b) it is the first RCT that will compare the influence of exercise in normoxia with EH, associated or not with LCD, on the glycemic, lipid profile, body composition, blood pressure and cardiorespiratory parameters; (c) our hybrid intervention (EH + LCD) has not been tested together before or compared with other treatment modality (EH); (d) it is the first study to evaluate the effect of both aerobic and strength exercises in hypoxia associated with dietary control in patients with T2DM.

However, our study protocol also has several limitations: (a) the possibility for voluntary bias, with failure to follow the dietary plan, even with all previously mentioned adherence strategies, which may lead to the inclusion of patients with higher carbohydrate intake than planned, although this may only affect the external validity of the study, but not its internal validity; (b) the exclusion criteria cover several comorbidities that are likely to prevail in patients with T2DM, such as dyslipidemia, high blood pressure, stroke or myocardial infarction. While this may enhance the generalizability of our results, individuals with these conditions may also benefit from interventions; (c) the apparently narrow hemoglobin A1c range (6.5%–10%) used as an inclusion criterion may also limit the generalizability of our results. Still, we wish to minimize the effect of the large reduction in basal glycemic as a potential contributor to any observed improvements in cardiovascular, metabolic and anthropometric parameters; (d) given its relatively short duration, the study is unlikely to assess clinical outcomes for preventing chronic complications associated with T2DM, such as myocardial infarction or atherosclerosis; (e) it may be difficult to make a difference in hemoglobin A1c change between the EH and intervention groups because the diet in this study is slightly carbohydrate restricted (40% of total energy intake).

The use EH isolated or combined with an LCD, may lead to a better understanding of their mechanism of action, with possible clinical implications in improving the quality of life of patients with T2DM, by delaying the onset of chronic complications, and reduce health costs. In order words, under usual circumstances, the results of this study may stimulate further creation of hypoxia chambers capable of simulating high altitude environments, and be a potential non-pharmacological therapeutic strategy. Such information could have applicability in the promotion of public health.

Briefly, this will be the first RCT to assess the impact of EH with or without an LCD on glycemic control, HIF1-α, body composition and cardiovascular risk factors in patients with T2DM. This study will not only help fill a significant research gap but also contribute to the growing field of nutrition, exercise and public health. The results from this study may also help guide future research in this area and inform advice given by clinicians to this specific population.

## Supplemental Material

sj-pdf-1-nah-10.1177_02601060231190663 - Supplemental material for Combined low-carbohydrate diet and long-term exercise in hypoxia in type 2 diabetes: A randomized controlled trial protocol to assess glycemic control, cardiovascular risk factors and body composition Supplemental material, sj-pdf-1-nah-10.1177_02601060231190663 for Combined low-carbohydrate diet and long-term exercise in hypoxia in type 2 diabetes: A randomized controlled trial protocol to assess glycemic control, cardiovascular risk factors and body composition by Raquel Kindlovits, Ana C Sousa, João L Viana, Jaime Milheiro, Franklim Marques and Vitor H Teixeira in Nutrition and Health

## References

[bibr1-02601060231190663] Albouaini K Egred M Alahmar A , et al. (2007) Cardiopulmonary exercise testing and its application. Postgraduate Medical Journal 83(985): 675–682. 17989266 10.1136/hrt.2007.121558 PMC2734442

[bibr2-02601060231190663] American Diabetes A (2000) Diabetes mellitus and exercise. Diabetes Care 23(Suppl 1): S50–S54.

[bibr3-02601060231190663] Baena-Diez JM Penafiel J Subirana I , et al. (2016) Risk of cause-specific death in individuals with diabetes: A competing risks analysis. Diabetes Care 39(11): 1987–1995. 27493134 10.2337/dc16-0614

[bibr4-02601060231190663] Boule NG Haddad E Kenny GP , et al. (2001) Effects of exercise on glycemic control and body mass in type 2 diabetes mellitus: A meta-analysis of controlled clinical trials. JAMA 286(10): 1218–1227. 11559268 10.1001/jama.286.10.1218

[bibr5-02601060231190663] Bull FC Al-Ansari SS Biddle S , et al. (2020) World Health Organization 2020 guidelines on physical activity and sedentary behaviour. British Journal of Sports Medicine 54(24): 1451–1462. 33239350 10.1136/bjsports-2020-102955 PMC7719906

[bibr6-02601060231190663] Burtscher M Pachinger O Ehrenbourg I , et al. (2004) Intermittent hypoxia increases exercise tolerance in elderly men with and without coronary artery disease. International Journal of Cardiology 96(2): 247–254. 15262041 10.1016/j.ijcard.2003.07.021

[bibr7-02601060231190663] Chan AW Tetzlaff JM Gotzsche PC , et al. (2013) SPIRIT 2013 Explanation and elaboration: Guidance for protocols of clinical trials. BMJ 346: e7586. 10.1136/bmj.e7586 PMC3541470 23303884

[bibr8-02601060231190663] Cioffi I Marra M Pasanisi F , et al. (2021) Prediction of resting energy expenditure in healthy older adults: A systematic review. Clinical Nutrition 40(5): 3094–3103. 33288302 10.1016/j.clnu.2020.11.027

[bibr9-02601060231190663] Colberg SR Sigal RJ Yardley JE , et al. (2016) Physical activity/exercise and diabetes: A position statement of the American diabetes association. Diabetes Care 39(11): 2065–2079. 27926890 10.2337/dc16-1728 PMC6908414

[bibr10-02601060231190663] Davis NJ Tomuta N Schechter C , et al. (2009) Comparative study of the effects of a 1-year dietary intervention of a low-carbohydrate diet versus a low-fat diet on weight and glycemic control in type 2 diabetes. Diabetes Care 32(7): 1147–1152. 19366978 10.2337/dc08-2108 PMC2699720

[bibr11-02601060231190663] Desplanches D Hoppeler H Linossier MT , et al. (1993) Effects of training in normoxia and normobaric hypoxia on human muscle ultrastructure. Pflugers Archiv 425(3–4): 263–267. 8309787 10.1007/BF00374176

[bibr12-02601060231190663] Elhayany A Lustman A Abel R , et al. (2010) A low carbohydrate Mediterranean diet improves cardiovascular risk factors and diabetes control among overweight patients with type 2 diabetes mellitus: A 1-year prospective randomized intervention study. Diabetes, Obesity & Metabolism 12(3): 204–209. 10.1111/j.1463-1326.2009.01151.x 20151996

[bibr13-02601060231190663] Evert AB Dennison M Gardner CD , et al. (2019) Nutrition therapy for adults with diabetes or prediabetes: A consensus report. Diabetes Care 42(5): 731–754. 31000505 10.2337/dci19-0014 PMC7011201

[bibr14-02601060231190663] Faramoushi M Amir Sasan R Sari Sarraf V , et al. (2016) Cardiac fibrosis and down regulation of GLUT4 in experimental diabetic cardiomyopathy are ameliorated by chronic exposures to intermittent altitude. Journal of Cardiovascular and Thoracic Research 8(1): 26–33. 27069564 10.15171/jcvtr.2016.05 PMC4827136

[bibr15-02601060231190663] Gildea N McDermott A Rocha J , et al. (2021) Time course of changes in Vo2peak and O2 extraction during ramp cycle exercise following HIIT versus moderate-intensity continuous training in type 2 diabetes. American Journal of Physiology. Regulatory, Integrative and Comparative Physiology 320(5): R683–R696. 10.1152/ajpregu.00318.2020 33624548

[bibr16-02601060231190663] Goldenberg JZ Day A Brinkworth GD , et al. (2021) Efficacy and safety of low and very low carbohydrate diets for type 2 diabetes remission: Systematic review and meta-analysis of published and unpublished randomized trial data. BMJ 372: m4743. 10.1136/bmj.m4743 PMC7804828 33441384

[bibr17-02601060231190663] Haider T Casucci G Linser T , et al. (2009) Interval hypoxic training improves autonomic cardiovascular and respiratory control in patients with mild chronic obstructive pulmonary disease. Journal of Hypertension 27(8): 1648–1654. 19387363 10.1097/HJH.0b013e32832c0018

[bibr18-02601060231190663] Jenkins DJA Kendall CWC Lamarche B , et al. (2018) Nuts as a replacement for carbohydrates in the diabetic diet: A reanalysis of a randomised controlled trial. Diabetologia 61(8): 1734–1747. 29789878 10.1007/s00125-018-4628-9 PMC6061153

[bibr19-02601060231190663] Joslin EP (1916) The treatment of diabetes Mellitus. Canadian Medical Association Journal 6(8): 673–684. 20310820 PMC1584654

[bibr20-02601060231190663] Kelsey MD Nelson AJ Green JB , et al. (2022) Guidelines for cardiovascular risk reduction in patients with type 2 diabetes: JACC guideline comparison. Journal of the American College of Cardiology 79(18): 1849–1857. 35512864 10.1016/S0735-1097(22)02840-6 PMC8972581

[bibr21-02601060231190663] Kindlovits R Pereira A Sousa AC , et al. (2022) Effects of acute and chronic exercise in hypoxia on cardiovascular and glycemic parameters in patients with type 2 diabetes: A systematic review. High Altitude Medicine & Biology 23(4): 301–312. DOI: 10.1089/ham.2022.0029. 36036723

[bibr22-02601060231190663] Kirk JK Graves DE Craven TE , et al. (2008) Restricted-carbohydrate diets in patients with type 2 diabetes: A meta-analysis. Journal of the American Dietetic Association 108(1): 91–100. 18155993 10.1016/j.jada.2007.10.003

[bibr23-02601060231190663] Krishnamachary B Berg-Dixon S Kelly B , et al. (2003) Regulation of colon carcinoma cell invasion by hypoxia-inducible factor 1. Cancer Research 63(5): 1138–1143. 12615733

[bibr24-02601060231190663] Ladage D Braunroth C Lenzen E , et al. (2012) Influence of intermittent hypoxia interval training on exercise-dependent erythrocyte NOS activation and blood pressure in diabetic patients. Canadian Journal of Physiology and Pharmacology 90(12): 1591–1598. 23210438 10.1139/y2012-138

[bibr25-02601060231190663] Lee PH Macfarlane DJ Lam TH , et al. (2011) Validity of the international physical activity questionnaire short form (IPAQ-SF): A systematic review. The International Journal of Behavioral Nutrition and Physical Activity 8: 115. 22018588 10.1186/1479-5868-8-115 PMC3214824

[bibr26-02601060231190663] Li J Li SX Gao XH , et al. (2019) HIF1A And VEGF regulate each other by competing endogenous RNA mechanism and involve in the pathogenesis of peritoneal fibrosis. Pathology Research and Practice 215(4): 644–652. 30598338 10.1016/j.prp.2018.12.022

[bibr27-02601060231190663] Lundby C Calbet JA Robach P (2009) The response of human skeletal muscle tissue to hypoxia. Cellular and Molecular Life Sciences 66(22): 3615–3623. 19756383 10.1007/s00018-009-0146-8 PMC11115669

[bibr28-02601060231190663] Lyer NV Kotch LE Agani F , et al. (1998) Cellular and developmental control of O2 homeostasis by hypoxia-inducible factor 1 alpha. Genes & Development 12(2): 149–162. 9436976 10.1101/gad.12.2.149 PMC316445

[bibr29-02601060231190663] Maiorana A O'Driscoll G Goodman C , et al. (2002) Combined aerobic and resistance exercise improves glycemic control and fitness in type 2 diabetes. Diabetes Research and Clinical Practice 56(2): 115–123. 11891019 10.1016/s0168-8227(01)00368-0

[bibr30-02601060231190663] Mathew TM Sharma S (2022) High Altitude Oxygenation. Treasure Island, FL: StatPearls. 30969523

[bibr31-02601060231190663] McArdle PD Greenfield SM Rilstone SK , et al. (2019) Carbohydrate restriction for glycaemic control in Type 2 diabetes: A systematic review and meta-analysis. Diabetic Medicine 36(3): 335–348. 30426553 10.1111/dme.13862

[bibr32-02601060231190663] Millet GP Debevec T Brocherie F , et al. (2016) Therapeutic use of exercising in hypoxia: Promises and limitations. Frontiers in Physiology 7: 224. 27375500 10.3389/fphys.2016.00224 PMC4902009

[bibr33-02601060231190663] Miyashita Y Koide N Ohtsuka M , et al. (2004) Beneficial effect of low carbohydrate in low calorie diets on visceral fat reduction in type 2 diabetic patients with obesity. Diabetes Research and Clinical Practice 65(3): 235–241. 15331203 10.1016/j.diabres.2004.01.008

[bibr34-02601060231190663] Moher D Schulz KF Altman D , et al. (2001) The CONSORT statement: Revised recommendations for improving the quality of reports of parallel-group randomized trials. JAMA 285(15): 1987–1991. 11308435 10.1001/jama.285.15.1987

[bibr35-02601060231190663] Myers J Prakash M Froelicher V , et al. (2002) Exercise capacity and mortality among men referred for exercise testing. New England Journal of Medicine 346(11): 793–801. 11893790 10.1056/NEJMoa011858

[bibr36-02601060231190663] Norton KI (2018) *Kinanthropometry and Exercise Physiology* .

[bibr37-02601060231190663] Ogurtsova K Guariguata L Barengo NC , et al. (2022) IDF Diabetes Atlas: Global estimates of undiagnosed diabetes in adults for 2021. Diabetes Research and Clinical Practice 183: 109118. 34883189 10.1016/j.diabres.2021.109118

[bibr38-02601060231190663] Robach P Cairo G Gelfi C , et al. (2007) Strong iron demand during hypoxia-induced erythropoiesis is associated with down-regulation of iron-related proteins and myoglobin in human skeletal muscle. Blood 109(11): 4724–4731. 17311997 10.1182/blood-2006-08-040006

[bibr39-02601060231190663] Rodbard HW Blonde L Braithwaite SS , et al. (2007) American Association of Clinical Endocrinologists medical guidelines for clinical practice for the management of diabetes mellitus. Endocrine Practice 13(Suppl 1): 1–68. 17613449 10.4158/EP.13.S1.1

[bibr40-02601060231190663] Sainsbury E Kizirian NV Partridge SR , et al. (2018) Effect of dietary carbohydrate restriction on glycemic control in adults with diabetes: A systematic review and meta-analysis. Diabetes Research and Clinical Practice 139: 239–252. 29522789 10.1016/j.diabres.2018.02.026

[bibr41-02601060231190663] Silverii GA Botarelli L Dicembrini I , et al. (2020) Low-carbohydrate diets and type 2 diabetes treatment: A meta-analysis of randomized controlled trials. Acta Diabetologica 57(11): 1375–1382. 32638087 10.1007/s00592-020-01568-8

[bibr42-02601060231190663] Snorgaard O Poulsen GM Andersen HK , et al. (2017) Systematic review and meta-analysis of dietary carbohydrate restriction in patients with type 2 diabetes. Bmj Open Diabetes Research & Care 5(1): e000354. 10.1136/bmjdrc-2016-000354 PMC5337734 28316796

[bibr43-02601060231190663] Switula D (2000) Principles of good clinical practice (GCP) in clinical research. Science and Engineering Ethics 6(1): 71–77. 11273440 10.1007/s11948-000-0025-z

[bibr44-02601060231190663] Thangarajah H Vial IN Grogan RH , et al. (2010) HIF-1alpha dysfunction in diabetes. Cell Cycle 9(1): 75–79. 20016290 10.4161/cc.9.1.10371

[bibr45-02601060231190663] Urdampilleta A Gonzalez-Muniesa P Portillo MP , et al. (2012) Usefulness of combining intermittent hypoxia and physical exercise in the treatment of obesity. Journal of Physiology and Biochemistry 68(2): 289–304. 22045452 10.1007/s13105-011-0115-1

[bibr46-02601060231190663] van Zuuren EJ Fedorowicz Z Kuijpers T , et al. (2018) Effects of low-carbohydrate- compared with low-fat-diet interventions on metabolic control in people with type 2 diabetes: A systematic review including GRADE assessments. American Journal of Clinical Nutrition 108(2): 300–331. 30007275 10.1093/ajcn/nqy096

[bibr47-02601060231190663] World Medical A (2013) World Medical Association Declaration of Helsinki: Ethical principles for medical research involving human subjects. JAMA 310(20): 2191–2194. 24141714 10.1001/jama.2013.281053

[bibr48-02601060231190663] Zhu Y Sidell MA Arterburn D , et al. (2019) Racial/ethnic disparities in the prevalence of diabetes and prediabetes by BMI: Patient outcomes research to advance learning (PORTAL) multisite cohort of adults in the U.S. Diabetes Care 42(12): 2211–2219. 31537541 10.2337/dc19-0532 PMC6868463

