# Supplementary material for: Combined low-carbohydrate diet and long-term exercise in hypoxia in type 2 diabetes: A randomized controlled trial protocol to assess glycemic control, cardiovascular risk factors and body composition
Source: Nutr Health. 2023 Jul 27;30(1):5–13. doi: 10.1177/02601060231190663 (PMC10924702; doi:10.1177/02601060231190663)
Supplement: sj-pdf-1-nah-10.1177_02601060231190663 - Supplemental material for Combined low-carbohydrate diet and long-term exercise in hypoxia in type 2 diabetes: A randomized controlled trial protocol to assess glycemic control, cardiovascular risk factors and body composition [file sj-pdf-1-nah-10.1177_02601060231190663.pdf]

U. PORTO

FACULDADE DE CIÊNCIAS DA NUTRIÇÃO E ALIMENTAÇÃO
UNIVERSIDADE DO PORTO

ETHICS COMMITTEE

OPINION No. 45/2021 / CEFCNAUP / 2021

Project title:

"Isolated and combined effect of a low carbohydrate diet and chronic exercise exposure to hypoxia on glycaemic control and cardiovascular risk factors in patients with type 2 diabetes".

Submitted by:

Raquel Kindlovits

Institutions involved in the study:

Clínica Médica de Exercício do Porto (CMEP), *Instituto Universitário da Maia* (ISMAI), *Faculdade de Ciências da Nutrição e Alimentação* (FCNAUP).

Rapporteur:

Teresa Amaral

Framework

This statement request refers to a study to be carried out within the scope of the Doctoral Thesis of Raquel Kindlovits, who is currently a student of the Doctoral Program in Clinical Nutrition at FCNAUP and a FCT doctoral fellow.

Research team:

Raquel Kindlovits (Ph. D. Student)

Vitor Hugo Teixeira, FCNAUP (Supervisor and Principal Investigator)

Ana Catarina Miranda de Sousa, ISMAI (Co-supervisor)

João Luis Viana, ISMAI (Co-supervisor)

Study objectives

The applicants intend to carry out an experimental study which aims to understand the combined impact of a diet and exercise intervention on biomarkers and metabolomics profile, on body composition, and physical fitness. The hypothesis under study is that a combination of a low carbohydrate diet (LCD) and exercise in hypoxia (EH) in physically active type 2 diabetes patients (T2Dp) is associated with improved glycaemic control and cardiovascular risk factors.

For this purpose, patients will be randomly assigned to three groups with ten participants per group: (1) control diet - low-fat and moderate-carbohydrate diet + exercise in normoxia; (2) EH group: control diet + EH; (3) group LCD + EH.

Volunteer patients will be recruited through the *"Diabetes em Movimento"* program and will do exercises that are already get used to. The exercise sessions will last for one hour, three times a week for eight weeks.

During all tests there will be doctors and/or nurses available.

Relevance and design of the study

The rationale for this experimental study is justified in the study protocol sent to this Ethics Committee, where the methodological aspects are described. Some details that were missing in this protocol were later clarified by the researchers.

Benefit / risks

Education to follow a diet according to guidelines and an exercise program can be regarded as a benefit of this intervention, as well the participants´ knowledge regarding their health assessments. Also, benefits are expected from both interventions (diet and exercise) on glycaemic control and cardiovascular health of the participant.

Otherwise, some risks can be enumerated, regarding:

- hypoxia during exercise will simulate an altitude of 3000m (14.5% O_2) and will be achieved through a gas chamber located at the Exercise Medical Centre & Spa (CMEP, Porto);
- blood samples collections of ≅1500µL, in each assessment;
- body composition assessment through Dual-energy X-ray Absorptiometry with radiation of approximately 1 micro-Sieverts;
- It is possible that some discomforts arise, such as symptoms of acute malaise during exercise in hypoxia, namely headache, dizziness, nausea, or shortness of breath during exercise. It should also be mentioned the discomfort related to exercise, blood collection, body composition assessment and other assessments.

Respect for the freedom and autonomy of the research subject

The eligible participants will be individuals with diagnosis of type 2 diabetes for at least one year, glycosylated haemoglobin less than 10%, pharmacological regimen stabilized for at least three months, main complications of diabetes tracked and controlled (diabetic retinopathy, diabetic nephropathy, diabetic foot and main factors of cardiovascular risk), previous participation in supervised exercise programs in the last 6 months and smoking absence in the last 6 months. Type 2 diabetic patients will undergo a detailed medical evaluation to screen for relative or absolute contraindications to moderate to high intensity exercise, including a treadmill maximal exercise test.

Freedom and autonomy are safeguarded in the information provided to the participant, and in the informed consent form.

Data confidentiality

It is guaranteed by the researcher and is transmitted at the time of informing the participant and obtaining informed consent.

Obtaining informed consent

Participants will be informed of all procedures and will give written consent to participate in the study. Information documents for the participant and for obtaining informed consent are in accordance with internationally established ethical standards.

Damage compensation:

Not mentioned.

Continuation of treatment / Follow-up of identified problems:

If data with clinical relevance are found, these should be communicated to the participant together with a viable possibility of clinical follow-up.

Curriculum of the researcher and team:

The curricula, of the research supervisor Vitor Hugo Teixeira, Assistant Professor at FCNAUP as well as the other members of the research team, are adequate for the research purposes.

Conclusion

From the above, this research project is scientifically justified, and it is understood that the present project meets the necessary conditions to receive a favourable opinion from FCNAUP Ethics Committee.

Faculty of Nutrition and Food Sciences, University of Porto, 23rd July 2021,

The Rapporteur	The President
Assinado por : **TERESA MARIA DE SERPA PINTO FREITAS DO AMARAL**<br>Num. de Identificação: 06938005	Sara SP Rodrigues
Teresa Amaral	Sara Rodrigues
